# Fluralaner as a novel treatment for sarcoptic mange in the bare-nosed wombat (*Vombatus ursinus*): safety, pharmacokinetics, efficacy and practicable use

**DOI:** 10.1186/s13071-020-04500-9

**Published:** 2021-01-06

**Authors:** Vicky Wilkinson, Kotaro Takano, David Nichols, Alynn Martin, Roz Holme, David Phalen, Kate Mounsey, Michael Charleston, Alexandre Kreiss, Ruth Pye, Elizabeth Browne, Christina Næsborg-Nielsen, Shane A. Richards, Scott Carver

**Affiliations:** 1 School of Natural Sciences, University of Tasmania, Private Bag 55, Hobart, Tasmania Australia; 2 The University of the Sunshine Coast, 90 Sippy Downs Dr, Sippy Downs, QLD Australia; 3 Central Science Laboratory, University of Tasmania, Private Bag 74, Hobart, Tasmania Australia; 4 Cedar Creek Wombat Rescue Inc, PO Box 538, Cessnock, NSW Australia; 5 The University of Sydney, C01A, JI Shute, Camden, Sydney, NSW Australia; 6 Bonorong Wildlife Sanctuary, 593 Briggs Rd, Brighton, Tasmania Australia

**Keywords:** Fluralaner, Sarcoptic mange, *Sarcoptes scabiei*, Bare-nosed wombat, Safety, Pharmacokinetics, Efficacy

## Abstract

**Background:**

Sarcoptic mange causes significant animal welfare and occasional conservation concerns for bare-nosed wombats (*Vombatus ursinus*) throughout their range. To date, *in situ* chemotherapeutic interventions have involved macrocytic lactones, but their short duration of action and need for frequent re-administration has limited treatment success. Fluralaner (Bravecto®; MSD Animal Health), a novel isoxazoline class ectoparasiticide, has several advantageous properties that may overcome such limitations.

**Methods:**

Fluralaner was administered topically at 25 mg/kg (*n* = 5) and 85 mg/kg (*n* = 2) to healthy captive bare-nosed wombats. Safety was assessed over 12 weeks by clinical observation and monitoring of haematological and biochemical parameters. Fluralaner plasma pharmacokinetics were quantified using ultra-performance liquid chromatography and tandem mass spectrometry. Efficacy was evaluated through clinical assessment of response to treatment, including mange and body condition scoring, for 15 weeks after topical administration of 25 mg/kg fluralaner to sarcoptic mange-affected wild bare-nosed wombats (*n* = 3). Duration of action was determined through analysis of pharmacokinetic parameters and visual inspection of study subjects for ticks during the monitoring period. Methods for diluting fluralaner to enable ‘pour-on’ application were compared, and an economic and treatment effort analysis of fluralaner relative to moxidectin was undertaken.

**Results:**

No deleterious health impacts were detected following fluralaner administration. Fluralaner was absorbed and remained quantifiable in plasma throughout the monitoring period. For the 25 mg/kg and 85 mg/kg treatment groups, the respective means for maximum recorded plasma concentrations (C_max_) were 6.2 and 16.4 ng/ml; for maximum recorded times to C_max_, 3.0 and 37.5 days; and for plasma elimination half-lives, 40.1 and 166.5 days. Clinical resolution of sarcoptic mange was observed in all study animals within 3–4 weeks of treatment, and all wombats remained tick-free for 15 weeks. A suitable product for diluting fluralaner into a ‘pour-on’ was found. Treatment costs were competitive, and predicted treatment effort was substantially lower relative to moxidectin.

**Conclusions:**

Fluralaner appears to be a safe and efficacious treatment for sarcoptic mange in the bare-nosed wombat, with a single dose lasting over 1–3 months. It has economic and treatment-effort-related advantages over moxidectin, the most commonly used alternative. We recommend a dose of 25 mg/kg fluralaner and, based on the conservative assumption that at least 50% of a dose makes dermal contact, Bravecto Spot-On for Large Dogs as the most appropriate formulation for adult bare-nosed wombats.

**Graphical abstract:**

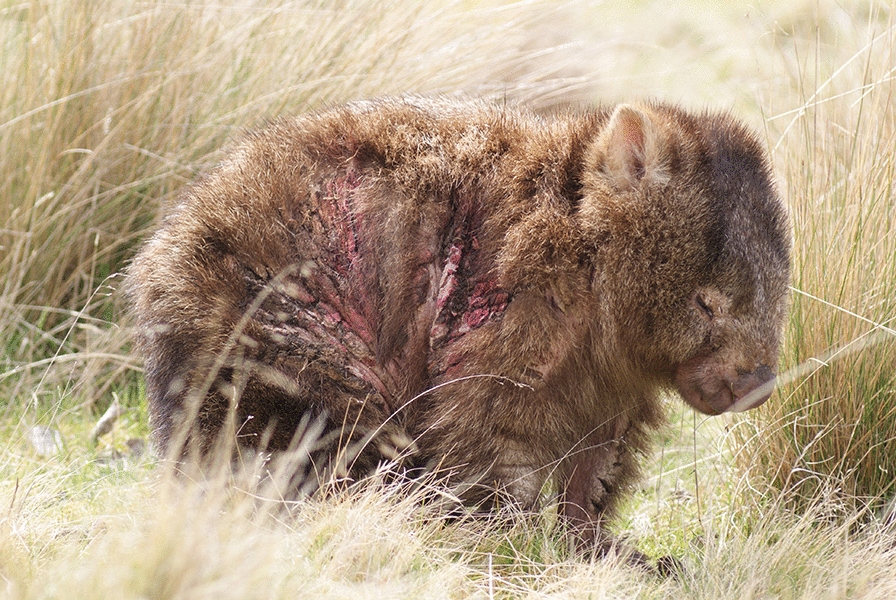

## Background

Establishing the safety, pharmacokinetic profile and efficacy of chemotherapeutic agents is a crucial step in the fight against infectious diseases that threaten wild animal health, conservation and welfare [1–5]. Despite this, veterinary drugs are often employed in the control of diseases in wildlife populations based on knowledge extrapolated from domestic animals, rather than through prior experimentation with target species [6–8]. Such pharmacological inference may result in treatment failure due to inefficacy or in undesirable adverse effects due to inter-species pharmacokinetic differences [8–10]. Sarcoptic mange (SM) is one such disease, for which numerous treatment strategies adapted from domestic animals have been attempted in wildlife, but where consensus on an evidenced-based, safe, efficacious and feasible solution for *in situ* disease control remains elusive [10, 11].

Caused by infection with *Sarcoptes scabiei,* an astigmatid mite that burrows into the host epidermis [12], SM has been recorded in over 100 species of wild mammals on multiple continents and is currently expanding in host and geographic range [9, 11, 13–17]. The first epizootic in free-living Australian wildlife was recorded in 1937 and involved bare-nosed wombats (*Vombatus ursinus*, hereafter BNWs) and introduced red foxes (*Vulpes vulpes*) from New South Wales [18]. Following initial emergence and spread, potentially facilitated by other mammals, such as foxes [19, 20], SM has become the most important infectious disease of the BNW, thought to be enzootic across the species’ range [20–23]. Intraspecies transmission is considered to occur predominantly by indirect contact through asynchronous usage of contaminated burrows [24–26], which represent an abiotic reservoir. This environmental source of infection, constituting all life stages of *S. scabiei* [27], forms a significant barrier to successful disease management [9, 12, 15, 28–32].

Bare-nosed wombats typically suffer from the most severe form of SM, termed parakeratotic mange (also known as crusted mange) [9, 17, 21], typified by highly visible clinical signs that include the progressive and sequential development of erythema, parakeratosis, alopecia, excoriation and weight loss [9, 11, 17, 33–36]. Due to its debilitating nature and protracted course, which can include survival of affected animals for several months post-infection [37], SM is primarily of animal welfare importance for BNWs [17, 38]. However, disease may also limit BNW population growth [39], and isolated epizootics have caused complete or near extirpation at the local level [15, 18].

The conspicuous disease presentation [36, 40] and well-documented impacts of localised SM epizootics [15, 18] have engendered widespread public concern for the welfare and conservation of BNWs [41] and increased the appetite for interventions in recent years [10, 41]. Thus, the scientific, government, wildlife rehabilitation and animal welfare communities have all participated in various chemotherapeutic disease control attempts, either at the individual level or population level [8, 9, 39, 41]. To date, this has most often involved administration of the macrocytic lactones (MLs), topical moxidectin [8, 38] or subcutaneous ivermectin [36, 42], to diseased BNWs. However, treatment success has proved highly variable and is limited by: (i) an absence of conclusive dose-determination studies and therefore an incomplete understanding of dose rates required to effectively treat varying degrees of SM in BNWs [10, 38, 41, 43]; (ii) pharmacokinetic and pharmacodynamic limitations, such as short durations of action and inefficacy against mite ova, that necessitate frequently repeated administration over several months [8, 10, 38, 41, 42]; and (iii), challenges associated with the direct delivery of drugs to wild animals, which are amplified by the need to re-treat the same individual on multiple occasions [8, 10, 38, 41, 43].

In light of these limitations, an important feature of feasible and successful SM management in free-living BNWs lies in the establishment of a safe therapeutic alternative that ensures clinical resolution following a single or small number of doses, and which also confers protection for long enough to eliminate environmental sources of infection [8, 10]. Furthermore, given the involvement of multiple stakeholders, an ideal therapeutic agent should be economical and easy to use in various situations [43]. Fluralaner (Bravecto^®^; MSD Animal Health, Merck & Co., Kenilworth, NJ, USA), a structurally unique isoxazoline class of ectoparasiticide [44], has recently been described as just such a treatment for SM in the dog [45, 46], cat (*Felis catus*) [47] and American black bear (*Ursus americanus*) [1]. With an apparent half-life of 12–15 days and detectable plasma levels for up to 112 days in dogs [48], a single fluralaner treatment has the potential to protect individual BNWs for long enough to break the life-cycle of *S. scabiei* in the environment, alleviate the need to regularly identify and re-treat individuals, be feasibly applied to BNWs in situ via the topical formulation [10] and reduce environmental exposure to pharmaceutical compounds. Thus, fluralaner represents a promising step towards achievable SM management in free-living BNWs [10, 49].

In this study, we aimed to: (i) test the safety and describe the pharmacokinetic profile of topical fluralaner in captive BNWs; (ii) evaluate the efficacy of topical fluralaner against naturally acquired *S. scabiei* infections in BNWs; (iii) establish a protocol for diluting fluralaner in its ‘spot-on’ formulation into a larger volume of suitable liquid for ‘pour-on’ application (enabling comparable application methods to MLs); and (iv) conduct an economic and treatment-effort analysis of fluralaner compared to moxidectin (the most-widely used alternative) for the treatment of SM in BNWs.

## Methods

### Safety and pharmacokinetics

One male and five female clinically healthy captive BNWs (3 juveniles and 3 adults), housed at Bonorong Wildlife Sanctuary (*n* = 4; denoted B1–4) and Zoodoo Wildlife Park (*n* = 2; denoted Z1–2), Tasmania, were enrolled in this study, as approved by the University of Tasmania’s Animal Ethics Committee (Approval Number: A16999). Two trials using ‘standard’ (25 mg/kg) and ‘high’ (85 mg/kg) fluralaner doses were conducted under veterinary supervision between August and October 2018, and December 2018 and February 2019, respectively. Routine monitoring of animals was conducted by researchers, facility staff, and the supervising veterinarian throughout the study periods, alongside supplementary behavioural monitoring undertaken by researchers (Appendix; Table 5).

During the 25 mg/kg trial (*n* = 5; B1–3, Z1–2), each BNW was anaesthetised for sampling followed by topical application of fluralaner to the interscapular epidermis on day 0, then anaesthesia and sampling were repeated on days 2, 4, 7, 9, 11, 14, 21, 28, 35, 49, 63, 77 and 91. The same protocol was followed for the 85 mg/kg trial (*n* = 2), excluding days 9, 11 and 28 to reduce anaesthetic burden on study animals. While one new BNW (B4) was included in the 85 mg/kg trial, additional animals could not be obtained. As such, animal availability and ethics permitted the re-use of one animal from the 25 mg/kg trial (B1). Anaesthesia was achieved by exposing animals to 5% isoflurane (Henry Schein Inc., Melville, NY, USA) at 3 l/min oxygen inside an induction chamber until recumbent, then 2–5% isoflurane at 2 l/min oxygen via facemask for maintenance. While anaesthetised, body weight was measured and 3 ml of blood was collected from the jugular vein with a 21-gauge needle. Cardiac and respiratory rates were measured and recorded every 5 min throughout anaesthesia until recovery.

From each blood sample, 1 ml was aliquoted into an ethylenediaminetetraacetic (EDTA) tube for haematological analysis, an EDTA tube for pharmacokinetic analysis and a lithium heparin tube for biochemical analysis. Samples intended for pharmacokinetic and biochemical analysis were centrifuged to separate plasma. Plasma for pharmacokinetic analysis was stored at − 80 °C in microcentrifuge tubes. All haematological and biochemical analyses were undertaken at IDEXX Laboratories (IDEXX Laboratories Pty. Ltd., Rydalmere, New South Wales, Australia) using a LaserCyte Dx Haematology Analyser and Catalyst Dx Chemistry Analyser, respectively (IDEXX Laboratories Pty, Ltd.). Samples were processed and submitted to the laboratory within 12 h of collection. Blood glucose measurements were excluded from analysis due to haemolysis-induced inaccuracies, and the results from days 35 and 63 of the 85 mg/kg trial were omitted due to delays in sample processing and analysis that resulted in significant haemolysis [50].

Changes in the haematological and biochemical values of BNWs in this study were assessed for clinical relevance and evidence of drug effects [51, 52]. Clinical relevance was defined as any haematological or biochemical change associated with clinical signs of ill health or as any value that deviated markedly from 95% reference intervals, and drug effect was defined as a parameter displaying a clear dose-response [51, 52]. Study-specific 95% reference intervals were calculated from values obtained on day zero (prior to fluralaner treatment) [51, 52], supported by results presented by Hartley et al. [40], Booth [53], Skerratt [36] and Ruykys et al. [42] for BNWs and the closely related southern hairy-nosed wombat (*Lasiorhinus latifrons*). Upper and lower interval limits were calculated as μ ± (1.96 × σ), where μ was the mean and σ the standard deviation of the mean. The mean, minimum and maximum values of each parameter obtained during the 25 mg/kg and 85 mg/kg trials were then compared against the intervals [51, 52].

Fluralaner plasma pharmacokinetics were assessed using ultra-performance liquid chromatography–tandem mass spectrometry (see Appendix 1 for details).

### Data analysis

Relationships between response variables (all measured haematological and biochemical parameters, body weight, plasma fluralaner concentration) and the predictor variable (number of days post-fluralaner administration) were analysed using generalised additive mixed models (GAMMs) [54]. In each model, a smoothing function was applied to the ‘Day’ variable and BNW ‘ID’ was used as a random effect to account for repeated measurements of the same individual. Separate GAMMs were run for each response variable, with animals grouped by dose rate, using the ‘mgcv’ package [54] in R [55]. For each dose rate, pharmacokinetic curves were plotted and standard pharmacokinetic parameters, comprising the maximum recorded plasma concentrations (C_max_) and times to C_max_ (T_max_), area under the curves (AUC), plasma elimination half-lives (t_1/2_) and mean residence times (MRT), were calculated using non-compartmental methods [56–58] with the ‘PK’ [59] package in R [55].

### Efficacy

Three free-living BNWs—one male juvenile (W1), one female juvenile (W2) and one male adult (W3)—exhibiting clinical signs of SM were captured in the Hunter Valley, New South Wales, and transported to Cedar Creek Wildlife Rescue and Hospital Inc., New South Wales, between July and November 2019. The animals were individually housed in purpose-built indoor enclosures containing straw bedding and an artificial dark shelter, simulating the burrow environment. Enclosure temperatures were monitored and maintained at < 20 °C. Food and water were provided *ad libitum* and straw bedding was replaced weekly.

Upon admission, a diagnosis of SM was made based on clinical observation, as described by Fraser et al. [60]. SM scores were then assigned to each BNW as per the methods of Simpson et al. [61]: briefly, a score of 0–10, based on the extent of SM clinical signs, was allocated to 14 body segments and the average of these represented the overall SM score. Body weight measurements and body condition scores were recorded [62], and SM was classified as mild, moderate or severe based on clinical evaluation of the above factors. BNWs were then visually inspected for the presence/absence of ticks to aid evaluation of fluralaner’s duration of action. Following assessment, 25 mg/kg fluralaner was applied to the interscapular epidermis.

Following fluralaner application on day zero, animals were monitored daily by an experienced wildlife rehabilitator or veterinarian throughout their time in captivity, with body weight measurements, body condition and SM scoring, and inspection for ticks repeated opportunistically as permitted by individual condition and behaviour, particularly aggression. Animals were transitioned to outdoor enclosures between weeks 8–12 post-treatment, where they would be exposed to naturally occurring ticks. Upon transfer to outdoor enclosures, monitoring was restricted to opportunistic scoring and monitoring for ticks until the end of the trial at 15 weeks post-treatment.

### Dilution

While it was possible to apply a topical ‘spot-on’ fluralaner preparation to the anaesthetised/restrained animals in the above safety, pharmacokinetic and efficacy trials, a larger volume of fluid that can be delivered as a ‘pour-on’ formulation (such as a spot-on pipette diluted into approximately 5 ml of additional fluid) is required for field administration. In preliminary trials, we evaluated several dilutant options, comprising canola oil mixed with acetone or d-Limonene or Orange Power Sticky Spot & Goo Dissolver (Aware Environmental Products, Dandenong South, VIC, Australia; hereafter Orange Power), and assessed the suitability of each formulation for field use by multiple stakeholders against the following criteria: relevant safety considerations listed in publicly available material safety data sheets; potential pharmacokinetic interaction(s) with fluralaner, based on known chemical properties and scientific literature; other relevant properties, such as acaricidal activity; time to chemical dissolution, determined by suspending 5 ml of each product in the contents of one Bravecto® Spot-On for Large Dogs pipette and observing time to dissolution (measured every hour for 12 h, then at 24, 36 and 48 h); odour; commercial availability; and cost per application. Although anecdotal reports suggest members of the public may be using unknown quantities of moxidectin as a dilutant, this was excluded from analyses because its safety for concurrent use in BNWs is unknown.

### Economic and treatment-effort analysis

The relative cost and effort associated with four SM treatment protocols for an adult BNW were compared. The protocols comprised: (i) a single application of Bravecto® Spot-On for Large Dogs (25 mg/kg fluralaner) with ≥ 6 ml dilutant, based on the expected duration of action derived from pharmacokinetic data [45, 46, 56] and efficacy trials (see Results); (ii) monthly applications of Bravecto® Spot-On for Large Dogs (25 mg/kg fluralaner) with ≥ 6 ml dilutant for 12 weeks, based on conservative interpretation of pharmacokinetic data (see Results); (iii) weekly application of 5 mL Cydectin® Pour-On for Cattle and Red Deer (Virbac Animal Health S.A., Carros, France; hereafter referred to as Cydectin) for 12 weeks (60 ml total, 0.2 ml/kg moxidectin), as per Martin et al. [8]; and (iv) weekly application of 20 ml Cydectin for 15 weeks (300 ml total, 0.8 ml/kg moxidectin), which was recently approved as a regime by the Australian Pesticides and Veterinary Management Authority (APVMA) [63]. Cost comparisons were made using the lowest commercial price found for each product online. Units of effort required to treat SM and prevent reinfection from environmental reservoirs [8] were also reported.

## Results

### Safety

Seven BNWs were treated with topical fluralaner, five with 25 mg/kg and two with 85 mg/kg, and no treatment-related effects on biochemistry (Table 1), haematology (Table 2), clinical condition or behaviour (Appendix; Table 5) were detected over the following 12 weeks. There was no substantive evidence of differences between the results obtained from the individual included in both trials and other study subjects. While significant temporal changes were observed in some clinical pathology parameters (Appendix; Figures 3, 4, 5, 6 and 7), all mean, minimum and maximum values remained within or close to reference intervals (Tables 1, 2) and there was no evidence of clinical relevance or association with a drug effect. Body weight increased significantly (*F*_1.5,1.5_ = 29.93,* P* < 0.001) over time but, similarly, this appeared to be independent of fluralaner administration.

**Table 1:** Biochemical reference intervals for healthy bare-nosed wombats (*Vombatus ursinus* and southern hairy-nosed wombats(*Lasiorhinus latifrons*) obtained from the literature together with the mean, minimum and maximum values obtained from healthy bare-nosed wombats at all time points during the 25 mg/kg and 85 mg/kg trials

Biochemical parameter	Reference intervals		25 mg/kg trial (*n* = 5)		85 mg/kg trial (*n* = 2)	
	BNW	SHNW	Mean	(Min–max)	Mean	(Min–max)
Sodium (mmol/l)	128.8–154.8	132.0–143.0	136.0^a^	129.0–146.0	136.9	135.0–140.0
Potassium (mmol/l)	1.4–17.1	4.1–10.8	5.4	4.0–8.4	5.4^a^	4.6–7.1
Chloride (mmol/l)	81.0–106.2	87.7–101.9	93.4	82.0–102.0	93.4	88.0–97.0
Bi-carbonate (mmol/l)	24.9–43.4	31.0–54.6	34.5^a^	27.0–47.0	32.2^a^	29.0–37.0
Sodium:potassium ratio	18.6–32.1	9.4–29.4	25.4^a^	*16.4*–*35.3*	25.7	19.0–29.8
Anion gap (mmol/l)	9.5–20.9	–	13.6^a^	*2.0*–*23.2*	16.7	12.5–19.6
Urea (mmol/l)	0.6–19.3	3.4–16.8	10.4	5.7–17.9	7.5	5.4–10.5
Creatinine (μmol/l)	34.0 – 278.8	72.4–307.6	80.0	40.0–130.0	91.0^a^	50.0–140.0
Phosphorous (mmol/l)	0.5–3.0	0.5–3.3	1.8	*0.9*–*4.5*	2.5^a^	*1.8*–*5.1*
Calcium (mmol/l)	1.9–3.4	2.3–2.7	2.6	*2.3*–*3.5*	2.5	2.3–2.7
Total protein (g/l)	46.1–86.5	54.5–72.5	62.2	57.0–70.0	58.8	54.0–64.0
Albumin (g/l)	20.2–38.2	22.0–43.0	34^a^	24.0–39.0	35.1	31.0–40.0
Globulin (g/lL)	16.9–53.6	17.0–37.8	28.2^a^	20.0–37.0	23.7	22.0–27.0
Albumin: globulin ratio	0.0–8.7	0.8–2.0	1.2^a^	0.7–1.9	1.5	1.2–1.7
Alkaline phosphatase (U/l)	0.0–1364.0	0.0–497.4	507.3^a^	242.0–884.0	920.7^a^	518.0–1339.0
Aspartate animotransferase (U/l)	0.0–186.8	4.0–73.8	60	42.0–111.0	59.8	41.0–100.0
Alanine aminotransferase (U/l)	0.0–162.6	0.4–93.0	35.2^a^	22.0–63.0	26.0	19.0–35.0
Creatine kinase (U/l)	0.0–5032.4	–	200.5	14.0–1593.0	463.3	26.0–2791.0
Cholesterol (mmol/l)	0.75–4.2	1.8–4.2	1.3	0.8–2.1	2.2^a^	1.4–2.9

Parameters that deviated from reference intervals for bare-nosed or southern-hairy nosed wombats are highlighted in italics

BNW, Bare-nosed wombat; min–max, minimum–maximum values; SHNW, southern hairy-nosed wombat

^a^Significant change observed over time-post fluralaner administration

**Table 2:** Haematological reference intervals for healthy bare-nosed and southern hairy-nosed wombats obtained from the literature together with the mean, minimum and maximum values obtained from healthy bare-nosed wombats at all time points during the 25 mg/kg and 85 mg/kg trials

Haematological parameter	Reference interval		25 mg/kg trial (*n* = 5)		85 mg/kg trial (*n* = 2)	
	BNW	SHNW	Mean	Min–max	Mean	Min–max
Red blood cells (×10^12^/l)	3.9–7.0	4.2–6.2	4.9	3.3–6.1	5.2	4.5–5.9
Haemoglobin (g/l)	77.6–168.6	108.8–163.6	123.2	80.0–153.0	124.2	113.0–136.0
Haematocrit (l/l)	0.3–0.5	–	0.4	*0.2*–*0.5*	0.4	0.3–0.4
Mean corpuscular volume (fl)	65.0–83.8	76.5–87.5	76^a^	70.0–86.0	70.4^a^	66.0–74.0
Mean corpuscular haemoglobin (pg)	12.7–32.3	24.1–28.9	25.4^a^	24.0–28.0	24.0^a^	22.0–26.0
Mean cell haemoglobin concentration (g/l)	288.7–377.1	299.3–343.9	333.8	303.0–357.0	340.9	321.0–358.0
White blood cells (× 10^9^/l)	0.9–19.7	0–18.7	9.1	4.7–15.3	8.6	6.3–11.2
Neutrophils (× 10^9^/l)	0.8–10.5	0–15.9	4.4	1.0–9.4	3.6	2.2–6.5
Lymphocytes (× 10^9^/l)	0.0–14.7	0–3.4	3.9	1.5–8.7	4.3	1.8–7.7
Monocytes (× 10^9^/l)	0.0–1.3	0–1.3	0.5	*0.0*–*2.1*	0.5	*0.0*–*1.6*
Eosinophils (× 10^9^/l)	0.0–1.7	0–1.5	0.3	0.0–1.1	0.2	0.0–0.4

Parameters that deviated from reference intervals for bare-nosed or southern-hairy nosed wombats are highlighted in italics

^a^Significant change observed over time-post fluralaner administration

At dose rates of 25 mg/kg and 85 mg/kg, mean corpuscular volume (25 mg/kg,* F*_2.5,2.5_ = 7.00,* P* = 0.001; 85 mg/kg,* F*_2.5,2.5_ = 7.00,* P* = 0.001) and mean corpuscular haemoglobin (25 mg/kg,* F*_2.1,2.1_ = 9.30,* P* < 0.001; 85 mg/kg,* F*_1.0,1.0_ = 21.57,* P* < 0.001) increased significantly over time (Appendix; Fig. 3), and alkaline phosphatase (ALP) decreased significantly (25 mg/kg, F_1.0,1.0_ = 26.42,* P* < 0.001; 85  mg/kg, F_1.9,1.9_ = 12.72, P < 0.001) (Appendix; Fig. 4). Bicarbonate also changed significantly over time at both dose rates, decreasing at 25 mg/kg (*F*_1.0,1.9_ = 8.21,* P* = 0.006) and increasing at 85 mg/kg (*F*_1.0,1.0_ = 6.52,* P* = 0.021) (Appendix; Fig. 4). Other significant changes observed at 25 mg/kg included increases in sodium (*F*_1.0,1.0_ = 14.08,* P* < 0.001), the sodium:potassium ratio (*F*_2.0,2.0_ = 5.16,* P* = 0.014), the anion gap (*F*_1.0,1.0_ = 12.02,* P* < 0.001), albumin (*F*_1.0,1.0_ = 14.9,* P* < 0.001), the albumin:globulin ratio (*F*_1.0,1.0_ = 11.03,* P* = 0.001), and alanine aminotransferase (*F*_1.0,1.0_ = 20.81,* P* < 0.001), and a decrease in globulin (F_1.0,1.0_ = 6.47, P = 0.013) (Appendix; Figs. 5, 6). At 85 mg/kg, significant increases were observed in potassium (*F*_1.0,1.0_ = 5.38,* P* = 0.034), creatinine (*F*_1.0,1.0_ = 48.59,* P* < 0.001) and phosphorous (F_3.5,3.5_ = 20.63, P ≤ 0.001), alongside a significant decrease in cholesterol (F_1.0,1.0_ = 4.90, P = 0.041) (Appendix; Fig. 7). No significant changes were observed across other parameters, and all values were within or comparable to reference intervals (Tables 1, 2).

**Fig. 1:**
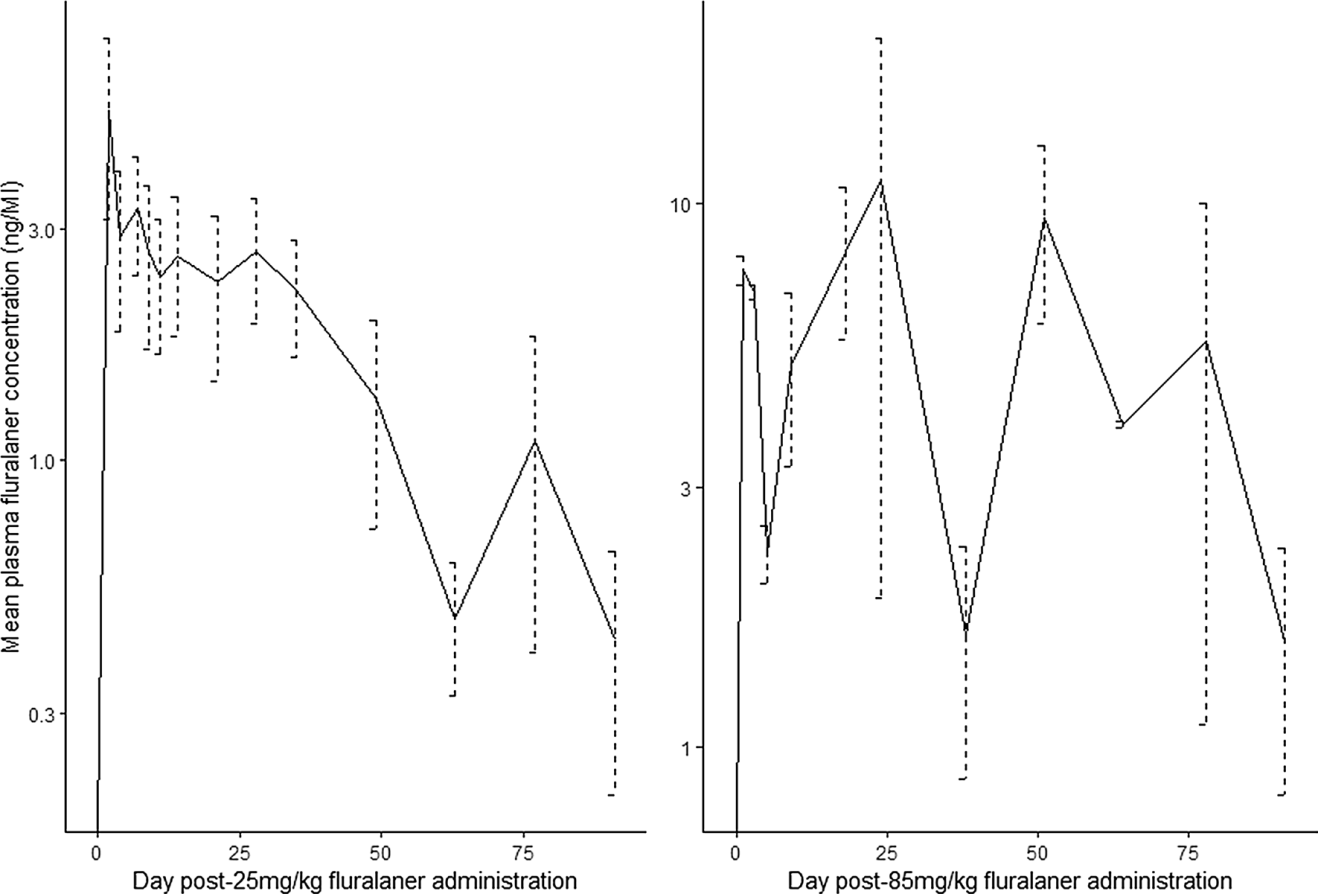
Mean and standard error fluralaner plasma concentrations in bare-nosed wombats following 25 mg/kg (*n* = 5) and 85 mg/kg (*n* = 2) administration

At both 25 mg/kg and 85 mg/kg, fluralaner was absorbed from the site of administration into the blood, where it remained quantifiable in plasma for over 12 weeks. However, there were marked differences between each dose rate’s pharmacokinetic profile (Fig. 1, Table 3). The mean C_max_ and mean AUC were 10.2 ng/ml and 363.9 day × ng/ml higher at 85 mg/kg than 25 mg/kg, and longer mean plasma persistence (T_1/2_ and MRT) was exhibited at 85 mg/kg (Table 4). Further, much greater fluctuations in plasma fluralaner concentration were observed at 85 mg/kg compared to 25 mg/kg (Fig. 1; Table 3).

**Table 3:** Mean and standard error of pharmacokinetic parameters following administration of 25 mg/kg (*n* = 5) and 85 mg/kg (*n* = 2) fluralaner to bare-nosed wombats (*Vombatus ursinus*)

Pharmacokinetic parameter	25 mg/kg		85 mg/kg	
	Mean	SE	Mean	SE
C_max_ (ng/ml)	6.2	1.7	16.4	3.6
T_max_ (day)	3	1	37.5	13.5
AUC (day*ng/ml)	152.9	18.4	516.8	119.9
T_1/2_ (day)	40.1	8.4	166.5	8.2
MRT (day)	32.0	3.4	46.8	8.3

**Table 4:** Economic and treatment-effort analysis of four sarcoptic mange treatment protocols for an adult bare-nosed wombat (*Vombatus ursinus*)

Product	Active ingredient	Dose	Volume (ml)	Administration frequency	Treatment duration (weeks)	Cost per application (AUD)^e^	Cost per animal (AUD)^e^	Treatment effort
Bravecto A^a^	Fluralaner	25 mg/kg	3.57	Once	12	27.55	27.79	1
+ OP^b^	N/A	N/A	≥ 6			0.24		
Bravecto B^a^	Fluralaner	25 mg/kg	3.57	Monthly	12	27.55	83.37	3
+ OP^b^	N/A	N/A	≥ 6			0.24		
Cydectin A^c^	Moxidectin	0.2 mL/kg	5	Weekly	12	1.09	13.19	12
Cydectin B^d^	Moxidectin	0.8 mL/kg	20	Weekly	15	4.40	65.99	15

Units of effort are relative to the total number of treatment applications required

^a^Bravecto Spot-on for Large Dogs

^b^Orange Power Sticky Spot & Goo Dissolver

^c^As per Martin et al. [8]

^d^As per regime recently approved by Australian Pesticides and Veterinary Management Authority

^e^At time of writing

### Efficacy

All three BNWs were diagnosed with SM, based on observable clinical signs of erythema, alopecia, epidermal crusting and pruritic dermatitis, and they were assigned initial mean SM scores of 1 (W1), 1.57 (W2) and 2.09 (W3). Body condition scores upon admission were 3 (W1 and W2, moderate) and 2 (W2, poor), and ticks were observed on all animals. Based on clinical appraisal of these findings, SM was deemed to be mild in W1 and W2 and moderate in W3.

**Fig. 2:**
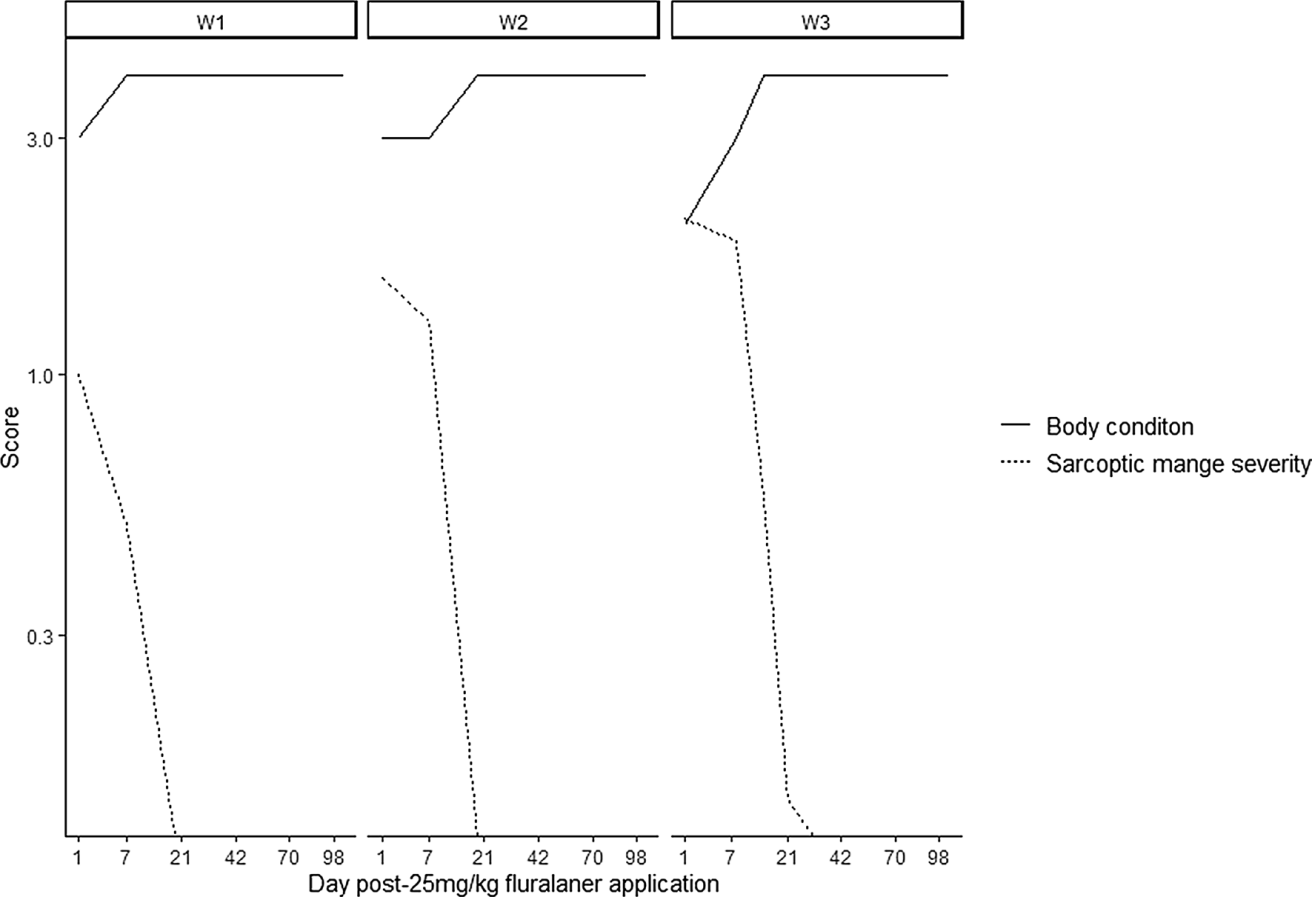
Sarcoptic mange and body condition scores for bare-nosed wombats over time post-25 mg/kg fluralaner administration

**Fig. 3:**
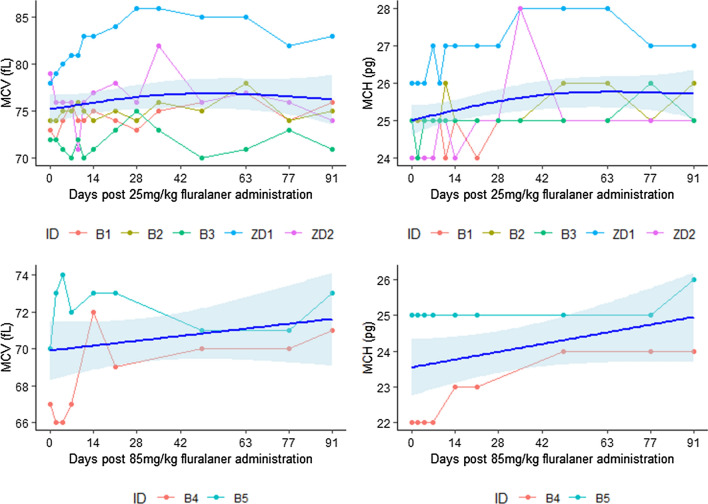
Observed (points and straight lines) and smoothed mean (curve) and standard error (shading) calculated by generalised additive mixed models (GAMM) for mean corpuscular volume (*MCH*, fl) and mean corpuscular haemoglobin (*MCH*, pg) in bare-nosed wombats (*Vombatus ursinus*) over time post 25 mg/kg and 85 mg/kg fluralaner administration

**Fig. 4:**
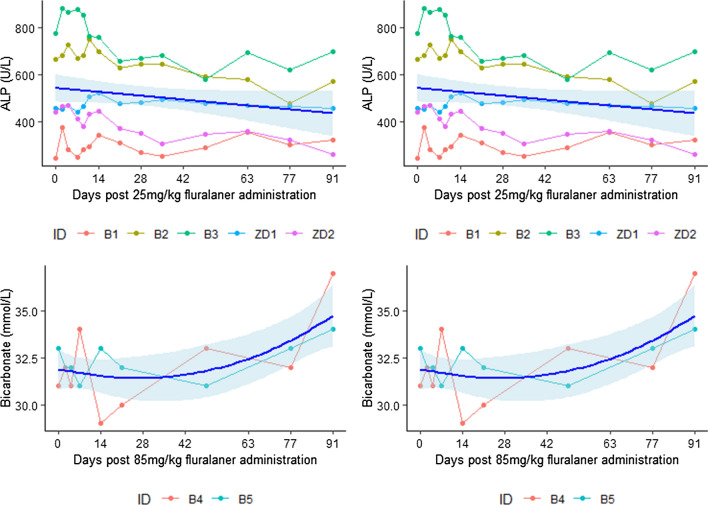
Observed (points and straight lines) and smoothed mean (curve) and standard error (shading) calculated by GAMM for alkaline phosphatase (*ALP*, U/l) and bicarbonate (mmol/l) in bare-nosed wombats over time post 25 mg/kg and 85 mg/kg fluralaner administration

**Fig. 5:**
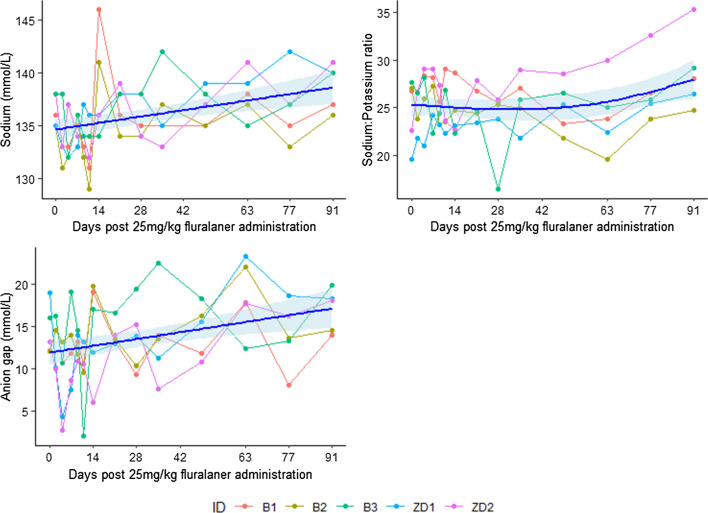
Observed (points and straight lines) and smoothed mean (curve) and standard error (shading) calculated by GAMM for the sodium (mmol/l), sodium:potassium ratio and anion gap (mmol/l) in bare-nosed wombats over time post 25 mg/kg fluralaner administration

**Fig. 6:**
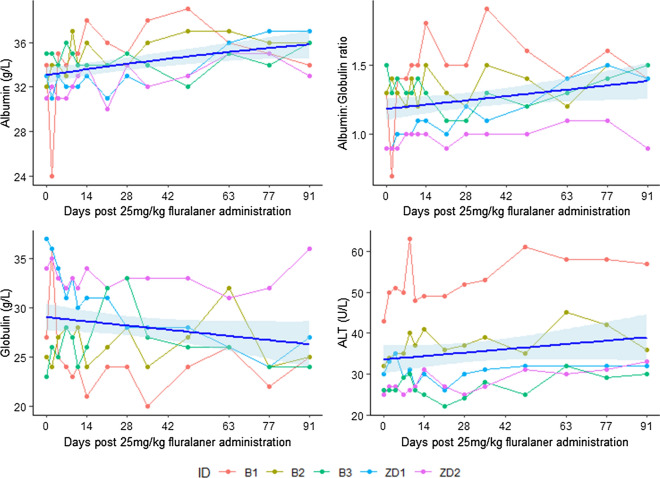
Observed (points and straight lines) and smoothed mean (curve) and standard error (shading) calculated by GAMM for albumin (g/l), albumin:globulin ratio, globulin (g/l) and alanine transaminase (ALT, U/l) in bare-nosed wombats over time post 25 mg/kg fluralaner administration

**Fig. 7:**
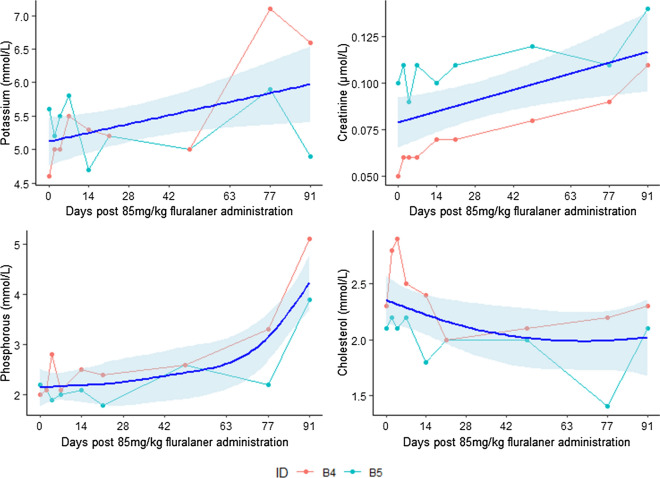
Observed (points and straight lines) and smoothed mean (curve) and standard error (shading) calculated by GAMM for potassium (mmol/L),creatinine (μmol/Ll, phosphorous (mmol/l) and cholesterol (mmol/l) in bare-nosed wombats over time post 85 mg/kg fluralaner administration

Following diagnosis and treatment with 25 mg/kg fluralaner, SM scores decreased by 50% (W1), 18% (W2) and 10% (W3) over the first 7 days, and by 100% (W1 and W2) and 72% (W3) over the first 3 weeks (Fig. 2). The SM score of W3 had decreased by 100% at 4 weeks post-treatment (Fig. 2). In concert with decreasing SM scores, all BNW body condition scores increased to 4 (optimum) within 2 weeks following fluralaner application and remained at that level for the duration of the study period (Fig. 2). All observable ticks had dropped off BNWs within the first week post-treatment and, despite having been in outdoor enclosures where ticks naturally occurred for the last 3 to 7 weeks of the trial, subjects remained tick free until 15 weeks post-treatment when monitoring ceased.

### Dilution

Orange Power was found to be the most favourable dilutant due its non-reactivity, similarity to the solvent present within Bravecto Spot-on products (Appendix; Table 6) lack of separation over 24 h, ready availability at major supermarkets, and comparatively low cost per application (Appendix; Table 6). Although other dilutant options tested did have some desirable qualities, several disadvantages of each rendered them less suitable overall (Appendix; Table 7).

### Economic and treatment-effort analysis

Upon comparing the cost and effort associated with two fluralaner and two moxidectin treatment regimes, our analysis indicated that 25  mg/kg fluralaner protocols were approximately 2.1-fold to 6.3-fold more expensive than the 0.2 ml/kg moxidectin protocol [8], but between 2.3-fold less expensive and 1.3-fold more expensive than the 0.8 ml/kg moxidectin protocol (Table 4). Owing to the expected long duration of efficacy determined by this study, we also estimated that using 25 mg/kg fluralaner to treat SM in a BNW requires 80–93% less effort than moxidectin (Table 4).

## Discussion

Sarcoptic mange negatively impacts the health and welfare of affected BNWs and other mammals worldwide [17, 39], causing local-level conservation issues on occasion [11]. Previous *in situ* chemotherapeutic attempts in BNWs have involved the use of MLs, but these efforts have suffered from numerous limitations [8, 41, 64]. Therefore, in order to manage the burden of disease, prevent and alleviate suffering and ensure better outcomes for BNWs with SM, there is need to establish a safe and efficacious long-acting chemotherapeutic agent for disease control [8].

We conducted rigorous safety, pharmacokinetic and efficacy trials on the isoxazoline class ectoparasiticide fluralaner (Bravecto®) [44] with a view to establishing a new gold standard for SM management in free-living BNWs. Our research showed that: (i) standard (25 mg/kg) and high (85 mg/kg) dose rates of fluralaner were safely tolerated by both juvenile and adult BNWs; (ii) fluralaner remained present at detectable quantities in BNW plasma for in excess of 90 days following administration at 25 mg/kg and 85  mg/kg; (iii) single 25 mg/kg doses were sufficient to ensure clinical resolution of mild and moderate SM in juvenile and adult BNWs, and provide tick prophylaxis for at least 1 month (based on T_1/2_) and likely up to 15 weeks (based on efficacy trials); (iv) Orange Power Sticky Spot & Goo Dissolver (Orange Power) could be safely, easily and economically used to dilute fluralaner into large enough volumes of liquid to be applied topically as a ‘pour-on’; and (v) both fluralaner protocols tested represented cost-competitive and effort-efficient treatment options relative to the most commonly used alternatives.

Fluralaner has been shown to have a wide safety margin across a broad taxonomic range of domestic and wild animal species [1, 51, 52, 65, 66], including at up to fivefold and 15-fold the recommended dose in dogs and chickens (*Gallus gallus domesticus*), respectively [51, 52]. The results presented here demonstrate that fluralaner can be safely administered to BNWs at up to 3.4-fold the minimum effective dose for dogs, such as at 25 mg/kg [46], and tolerance to the drug is expected to be similarly high in this species. While statistically significant changes in body weight and several clinical pathology parameters were observed in both the 25 mg/kg and 85 mg/kg trials, they were not associated with significant divergence from reference intervals [50, 52] nor was there evidence of clinical relevance or drug effects [51]. Although the precise cause(s) of these changes remain undetermined, fluctuations in haematological and biochemical values are commonly observed in nature, as well as under experimental conditions. For example, temporal variations in a range of parameters have been documented at the individual and group level in numerous free-living wild animal species, including marsupials, in response to climactic conditions, time of day, diet, stress, age and sampling procedure [50, 67–71]. Statistically significant but clinically irrelevant differences in clinical pathology parameters have also been documented between treatment and control groups of chickens and dogs during previous fluralaner safety trials [51, 52]. As such, the alterations observed in this study were considered to be physiological and most-likely related to intrinsic or extrinsic factors exclusive of fluralaner. Similarly, the observed increase in mean body weight was related to the growth of juvenile study subjects, rather than to any likely effect of drug administration.

Elucidating the pharmacokinetic profiles of 25 mg/kg and 85 mg/kg doses of fluralaner in BNWs confirmed that both dose rates, with T_1/2_ of > 40 days, may be sufficient to prevent reinfection while *S. scabiei* dies out in the local environment and to alleviate the need to re-identify and re-treat individual animals as regularly as for MLs [8, 38]. Relative to the persistence of moxidectin, which has a T_1/2_ of 5.03 days in SHNW [38], this represents a marked improvement in duration of action, dramatically reducing treatment effort and potential for treatment failure, while simultaneously increasing the feasibility of* in situ* SM control attempts.

Although the pharmacokinetic profile of topical fluralaner in BNWs differed notably from that of domestic dogs and cats [56], the differences were largely in favour of the former. For example, while the mean C_max_ documented in dogs administered 25 mg/kg fluralaner was > 700 ng/ml higher than we observed at the same dose, the T_1/2_ was approximately twofold longer in BNWs [56]. Similarly, at 20 mg/kg in cats, the mean C_max_ was > 700 ng/ml, but the mean T_1/2_ was just 13 days [56]. Despite the relatively short plasma persistence observed in companion animals, single administrations at these dose rates have consistently resulted in ectoparasitic prophylaxis for at least 3 months [72, 73]. As such, the comparatively long persistence of fluralaner in BNWs, which may arise from their metabolic rate being approximately only 40% of that of other mammals [74] or their relatively high levels of body fat [38], was expected to confer an equivalent or greater period of protection. Indeed, in combination with pharmacokinetic observations, the success of single 25 mg/kg fluralaner doses in preventing reinfection with *S. scabiei* and infection with ticks for the entire observation period suggested that the duration of action is likely in excess of 15 weeks in BNWs. Although challenge experiments, which remain a future direction of study, would have been required to definitively confirm duration of prophylaxis in this study, the pharmacokinetic and efficacy data reported here conservatively suggest that fluralaner is efficacious against *S. scabiei* in BNWs for at least 1 to 3 months, likely longer. Investigation into the absorption, concentration and persistence of fluralaner in BNW tissues, in particular skin and adipose, would contribute to a more complete understanding of efficacy and duration of action in this species, as well as interspecies pharmacokinetic differences [19, 56].

Reasons for the marked differences in C_max_ values between companion animals and BNWs are unknown, but likely relate to interspecies variation in drug absorption, distribution, metabolism and elimination [75]. While the mean C_max_ recorded at 25 mg/kg in this study was low in comparison to that of dogs and cats [56], this did not appear to impair the efficacy of this dose rate in BNWs. A single 25 mg/kg dose resulted in the rapid and complete clinical resolution of mild or moderate disease in all study subjects, indicating that fluralaner is a highly effective treatment for SM in this species. Smilar to the speed of clinical improvement seen in SM-affected canines treated with a minimum dose of 25 mg/kg fluralaner [45, 46], the SM scores of all three BNWs had reduced to zero by 19 (mild disease) to 30 (moderate disease) days post-treatment. In contrast, although experimental moxidectin efficacy data are lacking for free-living wildlife in general [76], efficacy trials involving repeated subcutaneous injections of 300 μg/kg ivermectin in BNWs did not result in resolution of clinical signs until 63 days post-treatment [36], representing an additional 33-44 days of animal welfare compromise and ongoing opportunity for mite deposition into environmental reservoirs.

At present, remote drug delivery techniques involving topical administration of ‘pour-on’ ectoparasiticides via ‘burrow flaps’ or a ‘pole and scoop’ are the most frequently employed and, often, best available means for SM treatment in free-living BNWs [8, 41]. Such non-invasive methods are considered advantageous over capture and the direct drug administration because: (i) SM treatment is predominantly carried out by members of the public with varying degrees of experience [41]; (ii) safe capture and restraint of wild animals requires high levels of expertise and equipment; and (iii) it is preferable to minimise treatment-associated stress that could further compromise the health of already debilitated animals [76–79]. However, there are currently no recommendations for fluralaner dilution, and concern exists over anecdotal reports of widespread inappropriate drug use involving the simultaneous application of fluralaner and moxidectin to SM-affected BNWs at varying dose rates. This unregulated practice violates APVMA permits and represents a risk to BNW health and welfare through the potential for harmful drug interactions [36, 76].

A priority of this study, therefore, was to establish a protocol for diluting fluralaner into a volume of liquid that was safe, chemically appropriate and commercially available at a low cost. We were satisfied that Orange Power met the stated requirements and surmised that its potential to cause mild skin irritation, although sub-optimal due to the pre-existence of dermal pathology in SM-affected animals, was comparable to that of topical medications used in SM treatment, fluralaner and moxidectin included, and acceptable given the degree of animal suffering caused by SM [17]. Further, its terpenoid ingredients likely confer fast-acting but short-lived acaricidal activity that may contribute to multi-modal ectoparasitic therapy without confounding the results of future *in situ* fluralaner efficacy trials. As such, although other options may come to light through forthcoming experimentation, Orange Power Sticky Spot and Goo Dissolver is our current recommendation for fluralaner dilution.

Due to the informal and frequently voluntary nature of current SM management efforts [41], treatment cost is an important consideration for the individuals and groups involved. On this basis, we report that fluralaner is competitive in its cost relative to moxidectin, particularly under the current APVMA permit [63]. Albeit beyond the scope of this study, inclusion of the additional labour costs involved with weekly applications of moxidectin for 12–15 weeks would further skew the results in favour of fluralaner use [80]. Similarly, in a resource-limited setting such as this, treatment effort is often of equal importance to price, and the low dosing requirement of fluralaner renders it by far the most effort-efficient option overall.

In common with research of this type, the use of both captive and free-living wild animals in this study imposed several methodological limitations. Firstly, sample sizes were small because they were subject to the low availability of healthy captive and SM affected wild BNWs. Also, although the potential impact(s) of fluralaner on reproductive success could not be evaluated in the absence of reproductively active adults and maternally dependent juveniles, several lines of evidence suggest that the standard and high dose rates of fluralaner used in this study are unlikely to pose a health risk to developing embryos or nursing young: (i) through experimental studies in rabbits, the European Medicines Agency set a no-observable adverse effect embryo toxicity limit of 10 mg/kg/day [81]; (ii) administration of fluralaner at threefold the maximum dose rate for dogs at 8-week intervals over 24 weeks until whelping (males) or weaning (females) did not impact reproductive function, puppy survival or number of puppies raised to weaning in comparison to control animals [81]; (iii) no adverse events were documented in puppies as young as 8 weeks old receiving doses up to fivefold the maximum (280 mg/kg) administered every 8 weeks for 16–24 weeks [52]; and (iv) a 25 mg/kg topical dose was well-tolerated in BNWs of pre-weaning age in this study. As such, while interspecies differences cannot be ruled out, the safety of repeated doses that grossly exceed the single standard dose of fluralaner for a BNW established by this study (25 mg/kg), alongside the similarity of fluralaner’s safety profiles for dogs and BNWs, suggest that the drug may also be used in the latter during all stages of reproduction, without ill effect.

Secondly, control groups were omitted on ethical grounds and considered unnecessary because fluralaner does not occur in nature. Similarly, animals involved in the safety trials were not subjected to post-mortem examination because, although this is common practice in laboratory-based studies [51], the euthanasia of healthy animals from zoological collections was unjustifiable and the clinical monitoring conducted was deemed sufficient. Thirdly, in relation to the pharmacokinetic trials, as captive animals would not submit to conscious blood draws, sampling time-points were spaced at intervals of at least 24 h to reduce anaesthetic burden. However, this may have lowered the likelihood of capturing the true C_max_ and T_max_ values. Additionally, although the reference intervals presented here were based on the best available data, this was sub-optimal in that they were derived from several small sample populations of predominantly adult animals from distinct geographic locations, for which differing sampling and analytical protocols were used [50].

Lastly, in lieu of laboratory testing, a diagnosis of SM for animals in the efficacy trial was based on clinical examination and SM scoring by an experienced veterinary nurse, under the supervision of a veterinary surgeon and appropriately skilled researchers [60]. An observational SM diagnosis has been frequently employed in situations involving free-living BNWs and other wild mammalian species [15, 82–84], and was considered preferable in the present study to minimise the imposition of additional stress and anaesthetic requirements on already debilitated and stressed wild animals for the purposes of sample collection [60, 77–79]. While this method has been found to under-diagnose early or sub-clinical cases, recent research involving BNWs has shown that the results of observational diagnoses correlate positively with microscopic examination of diagnostic skin scrapes [60], showing 57.14% sensitivity and 88.46% specificity. Further, sensitivity was found to be higher as the number of mites increased, suggesting that false negatives are of much greater concern than false positives [60]. As such, and taking into consideration the limited number of likely differential diagnoses and the positive response to targeted acaricidal treatment, particularly in the absence of adjunctive therapies such as antibiotic or antifungal agents, the diagnoses determined by clinical observation in this study are reported with sufficient confidence regarding their accuracy. However, future work in which fluralaner’s speed of kill [43] and mite reduction are quantified [85, 86], through microscopy or quantitative PCR [60], is planned and would undoubtedly complement the clinical observations described here.

Dose determination for the efficacy trial conducted as part of this study was based on extrapolation from other domestic and wild animal species [1, 45] and the wide safety margin demonstrated by our safety and pharmacokinetic trials. The 25 mg/kg dose rate was considered to be the most appropriate to account for the possibility that, when treatment is conducted *in situ* through relatively indiscriminate installation of burrow flaps [8, 41], individual animals may inadvertently be exposed to multiple doses when burrow sharing occurs [87]. Due to its success in treating the study subjects suffering from mild to moderate disease, we have established 25 mg/kg as a new standard dose for treating SM in BNWs. Although the small sample size available precluded greater examination of this dose rate’s efficacy across the entire spectrum of SM severity [19], the rapid clinical resolution achieved here, alongside promising anecdotal reports in BNWs and other species [1], suggest that this dose is also likely to be effective against more advanced disease. While this may be the case, it is important to note that decisions regarding treatment attempts in BNWs with severe SM should be made in consultation with a veterinary surgeon, and with consideration for: (i) the potential of morbidity factors such as secondary infections or systemic disease to result in treatment failure and/or death [46]; (ii) unacceptable animal welfare compromise; and (iii) the high transmission risk associated with heavy mite burdens [19]. As such, further clinical trials to improve knowledge of fluralaner’s efficacy and dosing requirements against all stages of SM would greatly benefit clinical decision-making [19].

## Conclusions

Fluralaner was found to be a safe and efficacious drug for the treatment of SM in BMW, holding significant advantages over macrocytic lactones in relation to speed and duration of efficacy, alongside ease and cost of treatment [43]. We add BNWs and a new taxonomic group to the growing list of those in which fluralaner has been successfully used to combat this debilitating disease, with implications for SM management in SHNW [42], the endangered northern hairy-nosed wombat (*Lasiorhinus krefftii*) and other Australian mammals, such as the koala (*Phascolarctos cinereus*) [88]. As such, the findings presented here signify an important advance in effective SM management, and we propose fluralaner as the agent of choice for future individual and population-scale control attempts in free-living BNWs.

## Data Availability

The datasets used and/or analysed during the current study are available from the corresponding author on reasonable request.

## Appendix

See Figs. 3, 4, 5, 6 and 7 and Tables 5, 6 and 7.

Fluralaner plasma pharmacokinetics were assessed using ultra-performance liquid chromatography-tandem mass spectrometry (UPLC-MS/MS) and a 9.1 mg/ml fluralaner solution (MSD Animal Health) as an intermediate standard. Calibration standards were then prepared in blank horse plasma, at concentrations of 182.4, 91.2, 45.6, 22.8, 11.4, 5.7, and 2.85 ng/ml. This was deemed suitable because comparison of external calibration curves prepared using BNW plasma and horse plasma demonstrated no difference in matrix effect and blank BNW plasma could not be obtained in sufficient quantities to prepare the required set of external calibration standards for each batch of samples. A 10-μl aliquot of a 400 ng/mL Fipronil Surrogate Standard was added to each calibration standard and plasma sample. Then 100 μl of plasma was spiked with 10 µl of Fipronil Surrogate Standard into an Eppendorf tube, which was sealed and vortexed to mix. The samples and calibration standards were then extracted with 400 µl of − 20 °C 1:1 (v/v) MeOH:acetone and stored at − 20 °C for 1 h before being transferred into a refrigerated centrifuge (4 °C) and spun at a relative centrifugal force of 21,130*g* for 10 min. The resulting supernatant was transferred to vials and blown to dryness under nitrogen. The extract was made up to a volume of 150 µl with 50% methanol. All solvents used were liquid chromatography grade.

**Table 5:** Behavioural monitoring data for bare-nosed wombats in the 25 mg/kg trial (B1, B2, B3, Z1 and Z2) and 85 mg/kg (B4 and B1a) fluralaner safety trials

ID	Age	Sex	Time	Day	Sleeping	Basking	Exploring	Feeding	Handling
B1	Adult	Female	1430–1630	− 1	12	0	0	0	0
B1			1040–1640	0	17	0	5	2	0
B1			1430–1630	1	10	2	0	0	0
B1			1430–1630	2	12	0	0	0	0
B1			1430–1630	3	12	0	0	0	0
B1			1430–1630	4	10	2	0	0	0
B1			1430–1630	7	12	0	0	0	0
B1			1430–1630	8	12	0	0	0	0
B1			1430–1630	9	12	0	0	0	0
B1			1430–1630	10	12	0	0	0	0
B1			1430–1630	11	12	0	0	0	0
B1			1430–1630	14	12	0	0	0	0
B1			1430–1630	17	12	0	0	0	0
B1			1430–1630	21	12	0	0	0	0
B1			1430–1630	24	12	0	0	0	0
B1			1430–1630	28	10	2	0	0	0
B1			1430–1630	31	12	0	0	0	0
B1			1430–1630	35	12	0	0	0	0
B1			1430–1630	38	12	0	0	0	0
B1			1430–1630	41	12	0	0	0	0
B1			1430–1630	48	12	0	0	0	0
B1			1430–1630	53	12	0	0	0	0
B1			1430–1630	60	12	0	0	0	0
B1			1430–1630	67	12	0	0	0	0
B1			1430–1630	81	12	0	0	0	0
B1			1430–1630	89	12	0	0	0	0
B2	Juvenile	Female	1430–1630	− 1	9	0	2	0	1
B2			1040–1640	0	10	0	6	7	1
B2			1430–1630	1	3	0	4	5	0
B2			1430–1630	2	1	0	4	6	1
B2			1430–1630	3	10	0	1	1	0
B2			1430–1630	4	8	0	0	4	0
B2			1430–1630	7	6	1	0	5	0
B2			1430–1630	8	0	0	4	5	3
B2			1430–1630	9	6	0	5	0	1
B2			1430–1630	10	4	0	0	7	1
B2			1430–1630	11	0	0	8	2	2
B2			1430–1630	14	6	3	0	3	0
B2			1430–1630	17	1	0	5	4	2
B2			1430–1630	21	6	0	3	1	2
B2			1430–1630	24	4	0	2	6	0
B2			1430–1630	28	10	0	0	2	0
B2			1430–1630	31	7	0	0	4	1
B2			1430–1630	35	4	0	3	3	2
B2			1430–1630	38	2	0	3	5	2
B2			1430–1630	41	4	0	0	7	1
B2			1430–1630	48	0	1	4	6	1
B2			1430–1630	53	3	1	5	1	2
B2			1430–1630	60	12	2	3	5	2
B2			1430–1630	67	0	0	8	3	1
B2			1430–1630	81	0	0	4	6	2
B2			1430–1630	89	3	3	1	3	2
B3	Juvenile	Female	1430–1630	− 1	10	0	0	0	2
B3			1430–1630	0	10	0	0	0	2
B3			1430–1630	1	9	0	0	1	2
B3			1430–1630	2	6	0	1	3	2
B3			1430–1630	3	10	0	0	1	1
B3			1430–1630	4	4	0	0	4	4
B3			1430–1630	7	10	0	0	0	2
B3			1430–1630	8	10	0	0	0	2
B3			1430–1630	9	3	0	2	2	5
B3			1430–1630	10	12	0	0	0	0
B3			1430–1630	11	12	0	0	0	0
B3			1430–1630	14	12	0	0	0	0
B3			1430–1630	17	6	0	1	0	5
B3			1430–1630	21	10	0	0	0	2
B3			1430–1630	24	7	0	0	2	3
B3			1430–1630	28	12	0	0	0	0
B3			1430–1630	31	10	0	0	0	2
B3			1430–1630	35	10	0	0	0	2
B3			1430–1630	38	7	0	1	2	2
B3			1430–1630	41	10	0	0	0	2
B3			1430–1630	48	12	0	0	0	0
B3			1430–1630	53	8	0	0	0	4
B3			1430–1630	60	12	0	0	0	0
B3			1430–1630	67	12	0	0	0	0
B3			1430–1630	81	6	1	2	1	2
B3			1430–1630	95	12	0	0	0	0
Z1	Adult	Female	1430–1630	− 1	7	2	3	0	0
Z1			1040–1640	0	4	0	2	6	0
Z1			1430–1630	1	10	0	0	2	0
Z1			1430–1630	2	7	0	0	5	0
Z1			1430–1630	3	10	0	0	2	0
Z1			1430–1630	4	8	0	0	4	0
Z1			1430–1630	7	10	0	0	2	0
Z1			1430–1630	8	12	0	0	0	0
Z1			1430–1630	9	10	0	0	2	0
Z1			1430–1630	10	11	0	0	1	0
Z1			1430–1630	11	12	0	0	0	0
Z1			1430–1630	14	9	0	2	1	0
Z1			1430–1630	17	12	0	0	0	0
Z1			1430–1630	21	11	0	0	1	0
Z1			1430–1630	24	12	0	0	0	0
Z1			1430–1630	28	12	0	0	0	0
Z1			1430–1630	31	11	0	0	1	0
Z1			1430–1630	35	12	0	0	0	0
Z1			1430–1630	38	11	0	0	1	0
Z1			1430–1630	52	10	0	1	1	0
Z1			1430–1630	66	12	0	0	0	0
Z1			1430–1630	80	12	0	0	0	0
Z2	Adult	Male	1430–1630	− 1	12	0	0	0	0
Z2			1040–1640	0	7	0	2	3	0
Z2			1430–1630	1	8	0	3	1	0
Z2			1430–1630	2	7	0	3	2	0
Z2			1430–1630	3	4	0	6	2	0
Z2			1430–1630	4	9	0	0	3	0
Z2			1430–1630	7	10	0	0	2	0
Z2			1430–1630	8	10	0	0	2	0
Z2			1430–1630	9	10	0	0	2	0
Z2			1430–1630	10	12	0	0	0	0
Z2			1430–1630	11	12	0	0	0	0
Z2			1430–1630	14	12	0	0	0	0
Z2			1430–1630	17	10	0	0	2	0
Z2			1430–1630	21	10	0	1	1	0
Z2			1430–1630	24	12	0	0	0	0
Z2			1430–1630	28	12	0	0	0	0
Z2			1430–1630	31	12	0	0	0	0
Z2			1430–1630	35	10	0	2	0	0
Z2			1430–1630	38	7	0	4	1	0
Z2			1430–1630	52	7	0	4	1	0
Z2			1430–1630	66	8	1	3	0	0
Z2			1430–1630	80	10	0	2	0	0
B4	Juvenile	Female	1300–1500	0	10	0	0	0	2
B4			1300–1500	2	11	0	0	0	1
B4			1300–1500	4	10	0	0	1	1
B4			1300–1500	7	11	0	0	0	1
B4			1300–1500	14	6	0	0	2	2
B4			1300–1500	21	8	2	0	1	1
B4			1300–1500	35	9	0	0	0	3
B4			1300–1500	49	11	0	0	0	1
B4			1300–1500	63	9	0	3	0	0
B4			1300–1500	77	10	0	0	0	2
B1a	Juvenile	Female	1300–1500	0	8	0	1	2	1
B1a			1300–1500	2	8	1	0	1	2
B1a			1300–1500	4	9	2	0	0	1
B1a			1300–1500	7	10	0	0	0	2
B1a			1300–1500	14	12	0	0	0	0
B1a			1300–1500	21	11	0	1	0	0
B1a			1300–1500	35	12	0	0	0	0
B1a			1300–1500	49	12	0	0	0	0
B1a			1300–1500	63	12	0	0	0	0
B1a			1300–1500	77	12	0	0	0	0

Following preparation, samples were analysed using a Waters Acquity H-Class UPLC instrument coupled to a Waters Xevo triple quadrupole tandem mass spectrometry (Waters Corp., Milford, MA, USA). A Waters Acquity UPLC BEH C18 column (2.1 mm × 100 mm × 1.7 µm) was used. The mobile phase consisted of two solvents: 0.1% (v/v) formic acid in water (solvent A) and acetonitrile (solvent B). The ultra performance liquid chromatography program was 80% to 5% A at 4 min, which was held for 2 min, followed by re-equilibration to starting conditions for 3 min. The flow rate was 0.35 ml min^−1^, the column was held at 35 °C, and the sample compartment at 6 °C. Sample injection volume was 10 µl. Approximate retention time for fluralaner was 4.6 min and fipronil was 4.3 min.

**Table 6:** Composition of Orange Power Sticky Spot & Goo Dissolver and Bravecto® Spot-On for Large Dogs

Bravecto® Spot-On for Large Dogs^a^		Orange Power Sticky Spot & Goo Dissolver^a^	
Ingredients	Concentration	Ingredients	Concentration
Fluralaner	280 mg/ml	Ethanol	300–600 mg/ml
Di-methyl acetamide	339 mg/ml	d-Limonene	300–600 mg/ml
Diethyltoluamide	100–200 mg/ml	Glycol ether	100–300 mg/ml
Acetone	100–200 mg/ml	Alcohols ethoxylated	< 100 mg/ml
“Non-hazardous ingredients”	Not specified	Citrus terpenes in orange peel oil	< 100 mg/ml

^a^As per safety data sheets

The mass spectrometer was operated in negative electrospray ionisation mode with a needle voltage of 3.0 kV, and multiple reaction monitoring (MRM) was used to detect the analytes. Quantitation of fluralaner was conducted using the MRM transition [M−H]− (*m*/*z*) 554.0 to (*m*/*z*) 534.0 with an optimised cone voltage of 44 V and collision energy of 23 V. Fipronil quantitation was achieved with the MRM transition (m/z) 434.9 to (m/z) 329.9 optimised with cone voltage of 43 V and collision energy of 23 V. Dwell time for each MRM transition was 120 ms. The ion source temperature was 130 °C, the desolvation gas was nitrogen at 950 l/h, the cone gas flow was 100 l/h, and the desolvation temperature was 450 °C.

**Table 7:** Evaluation of dilutant options

Dilutant(s)	Volume (ml)	Relevant safety considerations^b^	Pharmacokinetic interaction with fluralaner	Relevant properties	Time to separation (h)	Odour	Commercial availability	Cost per application (AUD)^c^
Canola oil	4	N/A	N/A	–	3	Relatively odourless	Supermarket	0.04
Acetone	1	N/A	Non-reactive (inert solvent)	–			Hardware	
d-Limonene	5	Skin irritation	Non-reactive (inert solvent)	Acaricidal	> 24	Citrus	Online purchase required	2.48
Orange Power^a^	5	Skin irritation	Non-reactive (inert solvent)	Acaricidal	> 24	Citrus	Supermarket	0.24

AUD, Australian dollar; N/A, Not available

^a^Orange power Sticky Spot & Goo Dissolver

^b^According to safety data sheet

^c^At time of writing

## References

[1] Van Wick M, Hashem B (2019). Treatment of sarcoptic mange in an American black bear (Ursus americanus) with a single oral dose of fluralaner. J Wildl Dis..

[2] Bernard RF, Grant EHC (2019). Identifying common decision problem elements for the management of emerging fungal diseases of wildlife. Soc Natur Resour..

[3] Tripp DW, Rocke TE, Streich SP, Abbott RC, Osorio JE, Miller MW. Apparent field safety of a raccoon poxvirus-vectored plague vaccine in free-ranging prairie dogs (*Cynomys *spp.), Colorado, USA. J Wildl Dis. 2015;51(2):401–10. 10.7589/2014-02-051 25588006

[4] Pedersen AB, Fenton A (2015). The role of antiparasite treatment experiments in assessing the impact of parasites on wildlife. Trends Parasitol..

[5] Bosch J, Sanchez-Tome E, Fernandez-Loras A, Oliver JA, Fisher MC, Garner TW (2015). Successful elimination of a lethal wildlife infectious disease in nature. Biol Lett..

[6] Leon-Vizcaino L, Cubero MJ, Gonzalez-Capitel E, Simon MA, Perez L, Rocio Ruiz deYbanez M, et al. Experimental ivermectin treatment of sarcoptic mange and establishment of a mange-free population of Spanish ibex. J Wildl Dis. 2001;37(4):775–85. 10.7589/0090-3558-37.4.775 11763741

[7] Kinzer HG, Meleney WP, Lange RE, Houghton WE (1983). Preliminary evaluation of ivermectin for control of Psoroptes ovis in desert bighorn sheep. J Wildl Dis..

[8] Martin AM, Richards SA, Fraser TA, Polkinghorne A, Burridge CP, Carver S (2019). Population-scale treatment informs solutions for control of environmentally transmitted wildlife disease. J Appl Ecol..

[9] Astorga F, Carver S, Almberg ES, Sousa GR, Wingfield K, Niedringhaus KD (2018). International meeting on sarcoptic mange in wildlife, June 2018, Blacksburg, Virginia, USA. Parasites Vectors..

[10] Rowe ML, Whiteley PL, Carver S (2019). The treatment of sarcoptic mange in wildlife: a systematic review. Parasites Vectors..

[11] Bornstein S, Mörner T, Samuel WM (2001). Sarcoptes scabiei and sarcoptic mange. Parasit Dis Wild Mamm.

[12] Arlian LG, Morgan MS (2017). A review of Sarcoptes scabiei: past, present and future. Parasites Vectors..

[13] Daszak P, Cunningham AA, Hyatt AD (2000). Emerging infectious diseases of wildlife–threats to biodiversity and human health. Science..

[14] Tompkins DM, Carver S, Jones ME, Krkosek M, Skerratt LF (2015). Emerging infectious diseases of wildlife: a critical perspective. Trends Parasitol..

[15] Martin AM, Burridge CP, Ingram J, Fraser TA, Carver S (2018). Invasive pathogen drives host population collapse: effects of a travelling wave of sarcoptic mange on bare-nosed wombats. J Appl Ecol..

[16] Alasaad S, Walton S, Rossi L, Bornstein S, Abu-Madi M, Soriguer RC (2011). Sarcoptes-world molecular network (Sarcoptes-WMN): integrating research on scabies. Int J Infect Dis..

[17] Martin AM, Fraser TA, Lesku JA, Simpson K, Roberts GL, Garvey J (2018). The cascading pathogenic consequences of Sarcoptes scabiei infection that manifest in host disease. R Soc Open Sci..

[18] Gray D (1937). Sarcoptic mange affecting wild fauna in New South Wales. Aust Vet J..

[19] Skerratt LF (2005). Sarcoptes scabiei: an important exotic pathogen of wombats. Microbiol Aust..

[20] Martin RW, Handasyde KA, Skerratt LF (1998). Current distribution of sarcoptic mange in wombats. Aust Vet J..

[21] Skerratt LF (2005). *Sarcoptes scabiei*: an important exotic pathogen of wombats. Microbiology Australia..

[22] Martin A, Skerratt L, Carver S. Sarcoptic mange in Australian wildlife. Fact Sheet for Wildlife Health Australia. https://wildlifehealthaustralia.com.au/FactSheets.aspx2017. https://wildlifehealthaustralia.com.au/FactSheets.aspx.

[23] Martin A, Carver S, Proft K, Fraser TA, Polkinghorne A, Banks S (2019). Isolation, marine transgression and translocation of the bare-nosed wombat (Vombatus ursinus). Evol Appl..

[24] Martin AM, Ricardo H, Tompros A, Fraser TA, Polkinghorne A, Carver S (2019). Burrows with resources have greater visitation and may enhance mange transmission among wombats. Aust Mammal..

[25] Evans MC (2008). Home range, burrow-use and activity patterns in common wombats (*Vombatus ursinus*). Wildl Res..

[26] Skerratt LF, Skerratt JHL, Banks S, Martin R, Handasyde K (2004). Aspects of the ecology of common wombats (*Vombatus ursinus*) at high density on pastoral land in Victoria. Aust J Zool..

[27] Arlian LG (1989). Biology, host relations, and epidemiology of Sarcoptes scabiei. Annu Rev Entomol..

[28] Soulsbury CD, Iossa G, Baker PJ, Cole NC, Funk SM, Harris S (2007). The impact of sarcoptic mange *Sarcoptes scabiei* on the British fox *Vulpes vulpes* population. Mammal Rev..

[29] Niedringhaus KD, Brown JD, Sweeley KM, Yabsley MJ (2019). A review of sarcoptic mange in North American wildlife. Int J Parasitol Parasites Wildl..

[30] Cypher BL, Rudd JL, Westall TL, Woods LW, Stephenson N, Foley JE (2017). Sarcoptic mange in endangered kit foxes (Vulpes macrotis mutica): case histories, diagnoses, and implications for conservation. J Wildl Dis..

[31] Iacopelli F, Fanelli A, Tizzani P, Berriatua E, Prieto P, Martinez-Carrasco C (2020). Spatio-temporal patterns of sarcoptic mange in red deer and Iberian ibex in a multi-host natural park. Res Vet Sci..

[32] Fraser TA, Charleston M, Martin A, Polkinghorne A, Carver S (2016). The emergence of sarcoptic mange in Australian wildlife: an unresolved debate. Parasites Vectors..

[33] Oleaga A, Casais R, Prieto JM, Gortazar C, Balseiro A (2012). Comparative pathological and immunohistochemical features of sarcoptic mange in five sympatric wildlife species in Northern Spain. Eur J Wildl Res..

[34] Walton SF, Currie BJ (2007). Problems in diagnosing scabies, a global disease in human and animal populations. Clin Microbiol Rev..

[35] Skerratt LF. Sarcoptic mange in the common wombat, Vombatus ursinus (Shaw, 1800). PhD thesis. Melbourne: Department of Veterinary Science, The University of Melbourne. 2001.

[36] Skerratt LF. Clinical response of captive common wombats (Vombatus ursinus) infected with* Sarcoptes scabiei* var.* wombati*. J Wildl Dis. 2003;39(1):179–92. 10.7589/0090-3558-39.1.179 12685082

[37] Newman TJ, Baker PJ, Harris S (2002). Nutritional condition and survival of red foxes with sarcoptic mange. Can J Zool..

[38] Death CE, Taggart DA, Williams DB, Milne R, Schultz DJ, Holyoake C (2011). Pharmacokinetics of moxidectin in the southern hairy-nosed wombat (Lasiorhinus latifrons). J Wildl Dis..

[39] Beeton NJ, Carver S, Forbes LK (2019). A model for the treatment of environmentally transmitted sarcoptic mange in bare-nosed wombats (Vombatus ursinus). J Theor Biol..

[40] Hartley M, English A. *Sarcoptes scabei var. wombati* infection in the common wombat (*Vombatus ursinus*). Eur J Wildl Res. 2005;51(2):117–21.

[41] Old JM, Sengupta C, Narayan E, Wolfenden J (2018). Sarcoptic mange in wombats—A review and future research directions. Transbound Emerg Dis..

[42] Ruykys L, Breed B, Schultz D, Taggart D (2013). Effects and treatment of sarcoptic mange in southern hairy-nosed wombats (Lasiorhinus latifrons). J Wildl Dis..

[43] Beugnet F, Liebenberg J, Halos L (2015). Comparative speed of efficacy against Ctenocephalides felis of two oral treatments for dogs containing either afoxolaner or fluralaner. Vet Parasitol..

[44] Ozoe Y, Asahi M, Ozoe F, Nakahira K, Mita T (2010). The antiparasitic isoxazoline A1443 is a potent blocker of insect ligand-gated chloride channels. Biochem Biophys Res Commun..

[45] Romero C, Heredia R, Pineda J, Serrano JA, Mendoza GD, Trapala P (2016). Efficacy of fluralaner in 17 dogs with sarcoptic mange. Vet Dermatol..

[46] Taenzler J, Liebenberg J, Roepke RK, Frenais R, Heckeroth AR. Efficacy of fluralaner administered either orally or topically for the treatment of naturally acquired* Sarcoptes scabiei* var.* canis* infestation in dogs. Parasites Vectors. 2016;9(1):392. 10.1186/s13071-016-1670-7 PMC4937584 27387742

[47] Curtis CF, Bourdeau PJ, Barr PA, Mukherjee R (2019). Use of the novel ectoparasiticide fluralaner in the treatment of feline sarcoptic mange. Vet Rec Case Rep..

[48] Kilp S, Ramirez D, Allan MJ, Roepke RK, Nuernberger MC (2014). Pharmacokinetics of fluralaner in dogs following a single oral or intravenous administration. Parasites Vectors..

[49] Martin AM, Richards SA, Fraser TA, Polkinghorne A, Burridge CP, Carver S. Population-scale treatment informs solutions for control of environmentally transmitted wildlife disease. J Appl Ecol. 2019;56(10):2363–75.

[50] Friedrichs KR, Harr KE, Freeman KP, Szladovits B, Walton RM, Barnhart KF (2012). ASVCP reference interval guidelines: determination of de novo reference intervals in veterinary species and other related topics. Vet Clin Pathol..

[51] Prohaczik A, Menge M, Huyghe B, Flochlay-Sigognault A, Traon GL (2017). Safety of fluralaner oral solution, a novel systemic antiparasitic treatment for chickens, in laying hens after oral administration via drinking water. Parasites Vectors..

[52] Walther F, Allan M, Roepke Rainer K, Nuernberger M (2014). Safety of oral administration of flavored chewable tablets containing fluralaner, (Bravecto™), a novel systemic antiparasitic drug, in dogs after oral administration. Parasit Vectors..

[53] Booth R. Wombats: care and treatment of sick, injured and orphaned animals.In: Dryden DI, editor. Wildlife in Australia—healthcare and management. Sydney: Post-graduate Foundation in Veterinary Science, University of Sydney; 1999. p. 1–10.

[54] Wood SN (2006). Generalized additive models: an introduction with R. Texts Stat Sci..

[55] R Core Team (2019). R: A language and environment for statistical computing.

[56] Kilp S, Ramirez D, Allan MJ, Roepke RK (2016). Comparative pharmacokinetics of fluralaner in dogs and cats following single topical or intravenous administration. Parasites Vectors..

[57] Walther FM, Allan MJ, Roepke RK (2015). Plasma pharmacokinetic profile of fluralaner (Bravecto) and ivermectin following concurrent administration to dogs. Parasites Vectors..

[58] Riegelman S, Collier P (1980). The application of statistical moment theory to the evaluation of in vivo dissolution time and absorption time. J Pharmacokinet Biopharm..

[59] Jaki T, Wolfsegger MJ (2011). Estimation of pharmacokinetic parameters with the R package PK. Pharm Stat..

[60] Fraser TA, Martin A, Polkinghorne A, Carver S (2018). Comparative diagnostics reveals PCR assays on skin scrapings is the most reliable method to detect Sarcoptes scabiei infestations. Vet Parasitol..

[61] Simpson K, Johnson CN, Carver S (2016). Sarcoptes scabiei: the mange mite with mighty effects on the common wombat (Vombatus ursinus). PLoS One..

[62] Jackson S. Australian mammals: biology and captive management. Clayton: CSIRO Publishing; 2007.

[63] Australian Pesticides and Veterinary Medicines Authority. Permit to allow supply and minor use of a registered agvet chemical: product for control of sarcoptic mange in wombats. Date of issue:15 June 2020. http://permits.apvma.gov.au/PER89040.PDF. Accessed 30 Nov 2020.

[64] Espinosa J, Perez JM, Raez-Bravo A, Fandos P, Cana-Manuel FJ, Soriguer RC (2020). Recommendations for the management of sarcoptic mange in free-ranging Iberian ibex populations. Anim Biodiv Conserv..

[65] Fisara P, Guerino F, Sun F (2018). Investigation of the efficacy of fluralaner spot-on (Bravecto®) against infestations of Ixodes holocyclus on cats. Parasit Vectors..

[66] Meadows C, Guerino F, Sun F (2017). A randomized, blinded, controlled USA field study to assess the use of fluralaner topical solution in controlling feline flea infestations. Parasites Vectors..

[67] Macgregor JW, Holyoake CS, Connolly JH, Robertson ID, Fleming PA, Warren KS (2017). A need for dynamic haematology and serum biochemistry reference tools: novel use of sine wave functions to produce seasonally varying reference curves in platypuses (Ornithorhynchus anatinus). J Wildl Dis..

[68] Hawley AW, Peden DG (1982). Effects of ration, season and animal handling on composition of bison and cattle blood. J Wildl Dis..

[69] Bhan C, Singh S, Hooda O, Upadhyay R, Beenam VM, Mangesh V. Influence of temperature variability on physiological, hematological and biochemical profile of growing and adult Sahiwal cattle. J Environ Res. 2012;7:2A.

[70] Rosenthal KL, Johnston MS, Shofer FS, Poppenga RH (2005). Psittacine plasma concentrations of elements: daily fluctuations and clinical implications. J Vet Diagn Invest..

[71] Rodríguez P, Tortosa FS, Gortázar C (2006). Daily variations of blood biochemical parameters in the red-legged partridge (Alectoris rufa). Eur J Wildl Res..

[72] Bosco A, Leone F, Vascone R, Pennacchio S, Ciuca L, Cringoli G (2019). Efficacy of fluralaner spot-on solution for the treatment of Ctenocephalides felis and Otodectes cynotis mixed infestation in naturally infested cats. BMC Vet Res..

[73] Laino MA, Cardinal MV, Enriquez GF, Alvedro A, Gaspe MS, Gurtler RE (2019). An oral dose of Fluralaner administered to dogs kills pyrethroid-resistant and susceptible Chagas disease vectors for at least four months. Vet Parasitol..

[74] Evans M, Green B, Newgrain K (2003). The field energetics and water fluxes of free-living wombats (Marsupialia: Vombatidae). Oecologia..

[75] Toutain P-L, Ferran A, Bousquet-Mélou A (2010). Species differences in pharmacokinetics and pharmacodynamics. Comparative and veterinary pharmacology.

[76] Rowe ML, Whiteley PL, Carver S (2019). The treatment of sarcoptic mange in wildlife: a systematic review. Parasites Vectors..

[77] Debrincat S, Taggart D, Rich B, Beveridge I, Boardman W, Dibben R. Effects of overnight captivity on antioxidant capacity and clinical chemistry of wild southern hairy-nosed wombats (*Lasiorhinus latifrons*). J Zool Wildl Med. 2014;45(3):469–75. 10.1638/2012-0154R.1 25314812

[78] Narayan EJ (2017). Evaluation of physiological stress in Australian wildlife: Embracing pioneering and current knowledge as a guide to future research directions. Gen Comp Endocrinol..

[79] Hing S, Narayan EJ, Thompson RCA, Godfrey SS (2016). The relationship between physiological stress and wildlife disease: consequences for health and conservation. Wildl Res..

[80] Hinkle NC, Jirjis F, Szewczyk E, Sun F, Flochlay-Sigognault A (2018). Efficacy and safety assessment of a water-soluble formulation of fluralaner for treatment of natural Ornithonyssus sylviarum infestations in laying hens. Parasites Vectors..

[81] Committee for Medicinal Products for Veterinary Use (CVMP). CVMP assessment report for Bravecto for spot-on solution for dogs and cats (EMEA/V/C/002526/X/0005). Amsterdam: European Medicines Agency. 2016. https://www.ema.europa.eu/en/documents/variation-report/bravecto-v-c-2526-x-0005-epar-assessment-report-variation_en.pdf.

[82] Cross PC, Almberg ES, Haase CG, Hudson PJ, Maloney SK, Metz MC (2016). Energetic costs of mange in wolves estimated from infrared thermography. Ecology..

[83] Pisano SRR, Zimmermann F, Rossi L, Capt S, Akdesir E, Burki R (2019). Spatiotemporal spread of sarcoptic mange in the red fox (Vulpes vulpes) in Switzerland over more than 60 years: lessons learnt from comparative analysis of multiple surveillance tools. Parasites Vectors..

[84] Scott DM, Baker R, Tomlinson A, Berg MJ, Charman N, Tolhurst BA (2020). Spatial distribution of sarcoptic mange (Sarcoptes scabiei) in urban foxes (Vulpes vulpes) in Great Britain as determined by citizen science. Urban Ecosystems..

[85] Beugnet F, de Vos C, Liebenberg J, Halos L, Larsen D, Fourie J (2016). Efficacy of afoxolaner in a clinical field study in dogs naturally infested with Sarcoptes scabiei. Parasite..

[86] Bernigaud C, Fang F, Fischer K, Lespine A, Aho LS, Mullins AJ (2018). Efficacy and pharmacokinetics evaluation of a single oral dose of afoxolaner against Sarcoptes scabiei in the porcine scabies model for human infestation. Antimicrob Agents Chemother..

[87] Evans MC (2008). Home range, burrow-use and activity patterns in common wombats (Vombatus ursinus). Wildl Res..

[88] Speight KN, Whiteley PL, Woolford L, Duignan PJ, Bacci B, Lathe S (2017). Outbreaks of sarcoptic mange in free-ranging koala populations in Victoria and South Australia: a case series. Aust Vet J..

