# Correction to: Fluralaner as a novel treatment for sarcoptic mange in the bare-nosed wombat (*Vombatus ursinus*): safety, pharmacokinetics, efficacy and practicable use

**DOI:** 10.1186/s13071-021-04658-w

**Published:** 2021-03-05

**Authors:** Vicky Wilkinson, Kotaro Takano, David Nichols, Alynn Martin, Roz Holme, David Phalen, Kate Mounsey, Michael Charleston, Alexandre Kreiss, Ruth Pye, Elizabeth Browne, Christina Næsborg-Nielsen, Shane A. Richards, Scott Carver

**Affiliations:** 1 School of Natural Sciences, University of Tasmania, Private Bag 55, Hobart, TAS Australia; 2 The University of the Sunshine Coast, 90 Sippy Downs Dr, Sippy Downs, QLD Australia; 3 Central Science Laboratory, University of Tasmania, Private Bag 74, Hobart, TAS Australia; 4 Cedar Creek Wombat Rescue Inc, PO Box 538, Cessnock, NSW Australia; 5 The University of Sydney, C01A, JI Shute, Camden, Sydney, NSW Australia; 6 Bonorong Wildlife Sanctuary, 593 Briggs Rd, Brighton, TAS Australia

## Correction to: Parasites Vectors (2021) 14:18 https://doi.org/10.1186/s13071-020-04500-9

Following publication of the original article [1], it was brought to our attention that the second author’s surname had been misspelled as ‘Tokano’ instead of ‘Takano’.

The name has since been updated in the original article and the (correct) name may be found in this correction.

The authors apologize for any inconvenience caused.
